# Inhomogeneous field in cavities of zero index metamaterials

**DOI:** 10.1038/srep11217

**Published:** 2015-06-16

**Authors:** Yangyang Fu, Yadong Xu, Huangyang Chen

**Affiliations:** 1 College of Physics, Optoelectronics and Energy & Collaborative Innovation Center of Suzhou Nano Science and Technology, Soochow University, No.1 Shizi Street, Suzhou 215006, China

## Abstract

In common media, electromagnetic wave always possesses a fluctuant field variation, analogous to an undulant surface of sea. While electromagnetic wave in the media with zero index metamaterials (ZIMs), whose refractive indices are near zero, homogeneous or constant field distribution will emerge, resembling a tranquil surface of lake. Such impression almost could be found in all previous literatures related to ZIMs. However, in this letter, we theoretically and numerically find that, in a cavity structure with ZIMs, when higher order modes (e.g., dipole modes) are excited inside cavity, inhomogeneous field could take place in ZIMs. Such a finding challenges the common perception in ZIMs: It is generally considered that homogeneous or constant field is generated in ZIMs. In addition, the proposed cavity structure herein could be used to manipulate radiation of light, such as enhancing or suppressing radiation, controlling radiation pattern and achieving isotropic or directive radiation, thereby potential applications are expected. These effects are well confirmed by numerical simulations.

Over the past years, there has been extensive research interest in metamaterials^1^,^2^,^3^,^4^. That is because metamaterials exhibit a much broader range of electromagnetic (EM) parameters which are not accessible in natural materials, and they have been successfully employed to achieve extraordinary optical phenomena^5^,^6^,^7^,^8^ and applications^9^,^10^,^11^,^12^. As a special kind of metamaterials, zero index metamaterials (ZIMs) recently also draw great attention, such as epsilon-near-zero (ENZ) metamaterials, mu-near-zero (MNZ) metamaterials and matched impedance zero index metamaterials (MIZIMs). Generally, the effective refractive indices of ZIMs by current technique almost are near zero, and the materials with identically zero index are meaningless in physics. Therefore, in the whole community, the materials with near zero indices are usually called ZIMs. In such media, as the refractive indices are near zero, EM wave will possess a small wave number, leading to a very diminutive phase variation (that is, homogeneous field). Due to such an intriguing property, ZIMs have been investigated extensively for various applications^13^,^14^,^15^,^16^,^17^,^18^,^19^,^20^,^21^,^22^,^23^,^24^,^25^,^26^,^27^,^28^,^29^, for instance, manipulating transmission in a ZIM waveguide with defects^13^,^14^,^15^,^16^, obtaining desired directive radiation or multi-beams^17^,^18^,^19^, enhancing radiation efficiency^20^,^21^,^22^, squeezing or bending EM wave in a sub-wavelength ENZ channel^23^,^24^,^25^, and unidirectional transmission^26^,^27^. To the best of our knowledge, in almost all related work, the field in ZIMs is regarded to be homogeneous owing to the intrinsic property of ZIMs. Recently, we found that additional higher order modes could be excited together with monopole mode in a ZIM waveguide with defects^28^. Sharing with the same spirit, here we suggest a cylindrical cavity structure with MNZ shell, and we will show that cavity modes will affect the field distribution in ZIMs. By insetting line source inside a cylindrical cavity with MNZ, regardless of the external shape, when the resonances of higher order modes (e.g., dipole modes) occur, they will induce inhomogeneous field in ZIMs, which will indeed call into question our common perception in ZIMs. Moreover, the proposed cavity structure could be used to manipulate radiation of light, to enhance or suppress radiation, to control radiation pattern and to achieve isotropic or directive radiation.

## Results

### Inhomogeneous field in near zero index metamaterials

**Figure 1:**
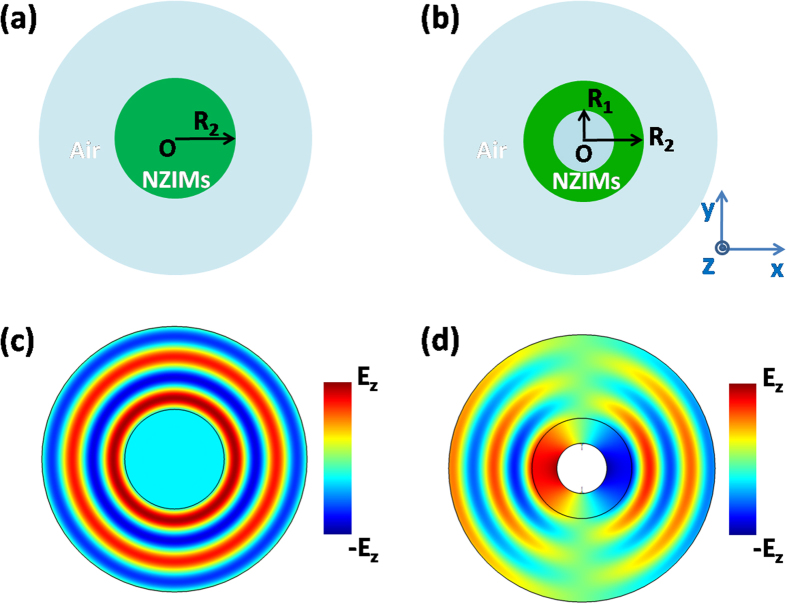
The homogeneous and inhomogeneous field in ZIMs. **(a)** The schematic plot of a cylinder of ZIMs placed in air. **(b)** The schematic plot of a cavity structure with ZIMs placed in air. The core medium is air as well. **(c)** The real part of electric field for a line source inserted in the ZIM cylinder. **(d)** The real part of electric field for a line source inserted in the cavity of ZIMs, where dipole mode is excited. For (**c**) and (**d**), the effective permittivity and permeability of MNZ are *ε*_2_ = 1 and *μ*_2_ = 10^−3^ respectively, and for MNZ shell, its inner and outer radii are *R*_1_ = 15 *mm* and *R*_2_ = 30 *mm*.

In general, the magnetic or electric field is almost homogeneous or constant in ZIMs. Before we start, let’s first match ZIMs with befitting polarization of EM wave. For MNZ metamaterials (e.g., *ε* = 1, *μ*→0), the effective polarization of EM wave is a transverse electric (TE) one. It can be deduced from 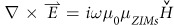. As *μ*_*ZIMs*_→0, to guarantee a finite value of magnetic field, 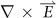 must vanish so that the electric field in ZIMs is a constant value (that is, homogeneous field). Likewise, for ENZ metamaterials (e.g., *ε*→0, *μ* *=* 1), the relevant polarization should be a transverse magnetic (TM) one. Without loss of generality, in this work, we will utilize MNZ metamaterials to verify the above discussion. For example, a cylinder of MNZ is placed in air as schematically shown in Fig. 1(a), and when a line source with TE polarization (electric field along *z* direction) is put inside this MNZ, we can observe homogeneous electric field in ZIMs as shown in Fig. 1 (c), which is consistent with common perception^18^,^20^,^23^. However, we discover that inhomogeneous field could happen in a ZIM cavity structure if higher order cavity modes are excited. Such a structure is composed of a core medium and a MNZ metamaterial cylindrical shell, with its inner and outer radii *R*_1_ and *R*_2_ respectively (see Fig. 1b). The effective permittivity and permeability of MNZ are *ε*_2_ and *μ*_2_ respectively. For the core region, we assume that it is filled with air. When a line source is put in the core region (but not at the center), and the dipole mode is excited inside the cavity, we can observe inhomogeneous field in ZIMs as shown in Fig. 1(d), where the electric field is decayed with an opposite phase in the left and the right sides of the core region. In the simulation, we set *R*_2_ = 2*R*_1_ = 30 *mm* for the cavity structure. As higher order modes are at resonance, quite intense magnetic field will take place in ZIMs so that the value of *μ*_2_*H* is not near zero, but becomes much larger. From 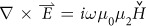, as 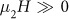, 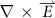 should not vanish. Therefore, the electric field is inhomogeneous and depends on the position, *i.e.*, 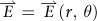. It seems that such result is contrary to our common perception, that is, there is homogeneous field with a constant phase in ZIMs. It is noted that MNZ metamaterials usually work for a narrow band of frequencies. In this work, we assume that MNZ metamaterials are dispersionless, in order to conveniently and clearly explain the potential physics of inhomogeneous field in the ZIM cavity structure.

### The influence of near zero permeability on the inhomogeneous field

**Figure 2:**
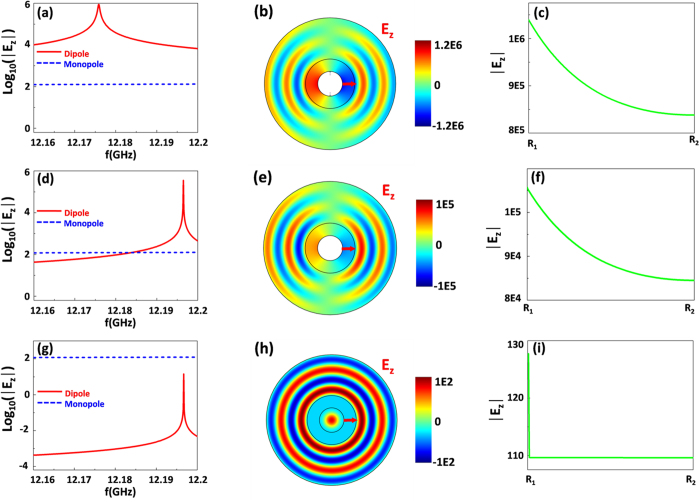
The influence of μ_2_ on the inhomogeneous field. **(a)**, **(d)** and **(g)** are the contrasts of electric field values between monopole mode (blue dashed curves) and dipole mode (red solid curves) for the case of *μ*_2_ = 10^−3^, *μ*_2_ = 10^−5^ and *μ*_2_ = 10^−10^ respectively. **(b)**, **(e)** and **(h)** are the real parts of electric field for dipole mode resonance for the case of *μ*_2_ = 10^−3^, *μ*_2_ = 10^−5^ and *μ*_2_ = 10^−10^ respectively. **(c)**, **(f)** and **(i)** are the electric field distributions from *R*_1_ to *R*_2_ for the case of *μ*_2_ = 10^−3^, *μ*_2_ = 10^−5^ and *μ*_2_ = 10^−10^ respectively. For MNZ shells, the related parameters are *R*_1_ = 15 *mm*, *R*_2_ = 30 *mm*, *ε*_2_ = 1. A line source with a current 1 A is positioned at the coordinate (10 *mm*, 0).

For the MNZ of cavity structure, the value of near zero permeability *μ*_2_ could greatly affect the field in ZIMs. For example, when it is strictly zero, the field of higher order modes will be trapped in the cavity^29^, *i.e*., the field of higher order modes could not exist in ZIMs. In our cavity structure, we should also address the influence of *μ*_2_ on the inhomogeneous field caused by the resonances of higher order modes. To make sense of this, we need to analyze electromagnetic field in ZIMs. We obtain the general solution *E*_*z*_ in ZIMs, which could be written as,
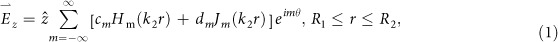
where *J*_*m*_(*x*) and *H*_*m*_(*x*)are the *m*-*th* order of Bessel function and Hankel function of the first kind respectively, 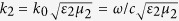 is the wave vector in MNZ, *ω* is angular frequency, *c* is the velocity of light in free space, *c*_*m*_ and *d*_*m*_ are the unknown coefficients to be determined. For the field in the core region and air, we express them in the Supplementary Note 1. By matching the boundary conditions at interfaces of *r* = *R*_1_ and *r* = *R*_2_, we can obtain the coefficients *c*_*m*_ and *d*_*m*_, as shown in Supplementary Note 1. In order to optimize Eq.(1), we define the ratio 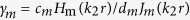. For *m* = 0, 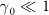, while for *m* ≠ 0, 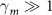 (see Supplementary Note 2), where we select *r* = *R*_1_ for *γ*_*m*_. Based on that, Eq.(1) could be approximately expressed as,
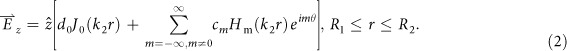


As we focus on the condition of higher order mode resonances, where the field of higher order mode is the most dominative term, Eq.(2) can be further simplified into such a form, which is the field superposition of monopole mode and higher order mode, as follows,
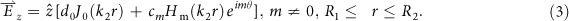
Here we choose the resonance of dipole mode as an example to illustrate the influence of *μ*_2_ on the inhomogeneous field. For dipole mode, the order *m* = −1 and *m* = 1 should be included in Eq.(3), hence the electric field in ZIMs is written as,
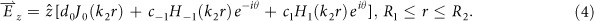
After simple calculation, it can be written as,
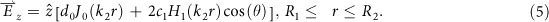


From Eq.(5), the total electric field is comprised of two components. One is the electric field of monopole mode *d*_0_*J*_0_(*k*_2_*r*), which is homogeneous field as *J*_0_(*k*_2_*r*)→1, while the other is the field of dipole mode 2*c*_1_*H*_1_(*k*_2_*r*)cos(*θ*), which is inhomogeneous field as it depends on the positions. To expediently compare the value of electric field of monopole mode with that of dipole mode in ZIMs, we just focus on the maximal values of both modes in the region of ZIMs, *i.e., d*_0_*J*_0_(*k*_2_*R*_*1*_) and 2*c*_1_*H*_1_(*k*_2_*R*_*1*_). If *μ*_2_ is equal to 10^−3^, when the working frequency is near the resonant one of dipole mode, Fig. 2(a) analytically shows the contrast result for the electric field values of monopole mode and dipole mode. We can find that the dipole mode is much more dominative than the monopole mode, thus the electric field in ZIMs is mainly composed of dipole mode and can be expressed approximately as 2*c*_1_*H*_1_(*k*_2_*r*)cos(*θ*), implying that the field in ZIMs is inhomogeneous. Numerically, when the resonance of dipole mode happens inside the cavity, the corresponding real part of electric field is shown in Fig. 2(b), where we can find that the field in ZIMs is inhomogeneous with an opposite phase in both sides of the core region. To clearly observe the inhomogeneous field in ZIMs, Fig. 2(c) numerically shows the electric field distribution from *R*_1_ to *R*_2_ (see the red arrow in Fig. 2b). It is obvious that the field in ZIMs is inhomogeneous as the field is damped from *R*_1_ to *R*_2_. Moreover, from the field distribution in Fig. 2(c), the maximal value of electric field in ZIMs is almost consistent with the theoretically approximate electric field value of dipole mode (see the peak of red curve in Fig. 2a). For the case of *μ*_2_ = 10^−5^, the contrast result of field values between monopole mode and dipole mode is analytically displayed in Fig. 2(d), where dipole mode is still dominative than monopole mode, but the value of electric field reduces slightly with the resonant frequency shifting a little bit. The corresponding field pattern and field distribution from *R*_1_ to *R*_2_ are represented in Fig. 2(e,f) respectively, where the inhomogeneous field still occurs in ZIMs. To be exact, we find that though the *μ*_2_ is a very tiny value *(i.e.,*10^−7^≤*μ*_2_≤10^−3^), in comparison to monopole mode, the dipole mode is more dominative, giving rise to the inhomogeneous field in ZIMs. However, when *μ*_2_ is extremely tiny (*i.e., μ*_2_≥10^−10^), e.g., 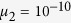, the monopole mode will be more dominative than dipole mode (see the analytical result in Fig. 2g). Hence, the electric field in ZIMs can be expressed approximately as *d*_0_*J*_0_(*k*_2_*r*), which is homogeneous field as *J*_0_(*k*_2_*r*) tends to 1 due to *k*_2_ → 0. The numerical simulation is also displayed in Fig. 2(h), where homogeneous field in ZIMs can be observed, which proves our analytical result well. Likewise, we numerically plot the electric field distribution from *R*_1_ to *R*_2_ as shown in Fig. 2(i). Obviously, the field in ZIMs is almost homogeneous though there is a sharp drop nearby the boundary *R*_1_. That is because when *μ*_2_ is extremely tiny, the MNZ shell will be opaque for higher order modes (e.g., dipole mode). While for monopole mode, it is penetrable. Therefore, the field from dipole mode will quickly vanish at the boundary *R*_1_, inducing such a sharp drop. Furthermore, the field value of monopole mode in Fig. 2(i) is about 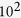, which matches well with the analytical result (see the blue dashed curve in Fig. 2g).

### Enhancing or suppressing radiation

In the above discussion, we have demonstrated that inhomogeneous field could occur in ZIMs in the above proposed cavity with MNZ metamaterials when the value of near zero parameter is not extremely tiny. For such cavity structure, it could be used to manipulate radiation of light, such as to enhance or to suppress radiation of light. Supplementary Fig. S3(c) and (d), analytically and numerically show the corresponding results of the power flow radiating from the cylindrical cavity respectively, where we can see both results are concurrent. There are a wealth of resonant peaks resulted from the resonance of each cavity mode, which is clarified by the order *m* one by one in the plot. When they are at the resonant frequencies of cavity modes, strong energy could radiate from the cavity to the air, which can be used to enhance radiation of light (see Supplementary Note 3). While for the frequencies deviating from such resonant frequencies, most energy will be confined inside the cavity so that it could be used to suppress radiation of light. By observing all peaks in Supplementary Fig. S3(c) and (d), we can find that the bandwidths of monopole modes are much broader, while the resonant peaks of higher order modes (*m* ≠ 0) are quite sharp. Such sharp resonances have great applications among optical sensors or detecting systems^30^,^31^.

### The influence on radiation with different MNZ

In fact, we consider a lossless MNZ metamaterials in the above analysis. However, for real ZIMs, their losses should be involved. Thereby, it is necessary to discuss the influence of losses on cavity resonances and the consequent radiated EM energy from MNZ cavity structure. In addition, the problem of the sensitivity of cavity resonances to near zero permeability 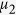 should also be addressed. For simplicity, we take the first peak of dipole mode (*m* = 1) in Supplementary Fig. S3 as an example, where the resonant frequency is about 12.167 GHz.

**Figure 3:**
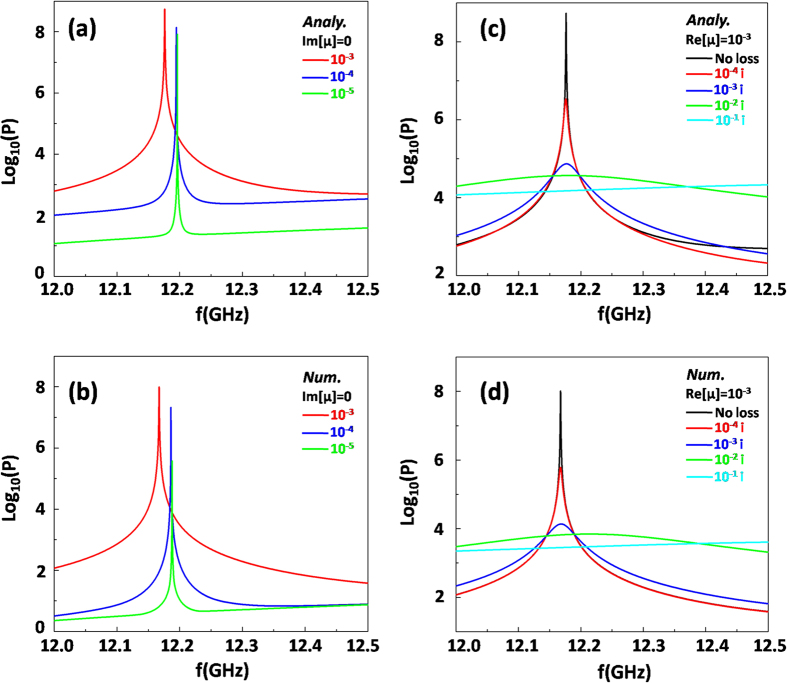
Influence on the radiation of dipole mode with different MNZ metamaterials. **(a)** and **(b)** are analytical and numerical results of radiated power flow for lossless cases respectively. The red, blue and green curves are for the case of *μ*_2_ = 10^−3^, 10^−4^ and 10^−5^ respectively. **(c)** and **(d)** are analytical and numerical results of radiated power flow for loss cases respectively. The black, red, blue, green and cyan curves are for the case of Im[*μ*_2_] = 0, 10^−4^, 10^−3^, 10^−2^ , 10^−1^ respectively, with a fixed Re[*μ*_2_] = 10^−3^. All the results are in a logarithmic scale. In all calculations, the related parameters are as follows: *R*_1_ = 15 *mm*, *R*_2_ = 30 *mm*, and *ε*_2_ = 1. The core media are air. A line source with a current 1 A is positioned at the coordinate (10 *mm*, 0).

For lossless case, Fig. 3(a) shows analytical results of power flow radiating from the cavity structure *vs* frequencies for different *μ*_2_. We can find that the resonant peak will slightly shift to a higher frequency as the value *μ*_2_ deceases from 10^−3^ to 10^−5^, and in such a changing process, the peak becomes much sharper. In consequence, we can realize distinctly that when the permeability tends to be smaller, it is more difficult for dipole mode to be excited out and for EM energy to radiate to free space. When the permeability is exactly zero, *i.e., μ*_2_ = 0, all EM energy of dipole mode will be localized inside the cavity completely, without any radiation. Moreover, Fig. 3(b) shows the numerical results of radiated power flow for comparison, which exhibits the consistent variation tendency with Fig. 3(a). Obviously, the numerical results agree with the analytical ones very well.

When the loss is involved, we take the case of Re[*μ*_2_] = 10^−3^  as an example to study concretely. The corresponding analytical results of different loss values are shown in Fig. 3(c), where the black, red, blue, green and cyan curves show the corresponding radiated power flow for different loss levels 0, 10^−4^, 10^−3^, 10^−2^ and 10^−1^ respectively. We find that the resonant peak becomes wider and the radiated power flow is weaker, with the loss increasing. Moreover, when the material loss reaches to 10^−1^ (see the cyan curve), the resonance peak almost disappears. Fig. 3(d) is the numerically calculated radiated power flow, which illustrates the same physics with theoretical results. To further quantify each resonant peak in Fig. 3(d), the quantity (Q) factor defined by 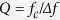 is figured out, where *f*_*c*_ is the resonant frequency, and Δ*f* is the full width at half maximum (FWHM). For losses from 0 to 10^–1^, the corresponding Q factor are numerically calculated as 30419, 2645, 284.3, 32.9, 0, respectively. Without doubt, the loss could greatly reduce the resonant effect of cavity modes and weaken the radiated EM energy.

### Controlling radiation pattern

**Figure 4:**
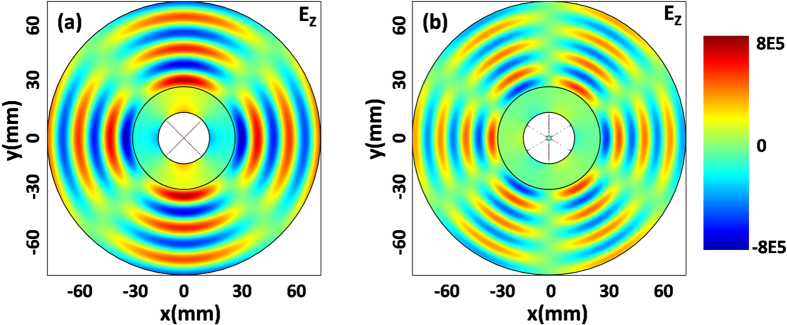
Simulated electric field patterns for higher order modes. **(a)** is the electric field pattern for cavity mode with *m* = 2. **(b)** is the electric field pattern for cavity mode with *m* = 3. In the simulations, the core media (region 3) are air. For MNZ shells (region 2), the parameters are *R*_1_ = 15 *mm*, *R*_2_ = 30 *mm*, *μ*_2_ = 10^−3^ and *ε*_2_ = 1. A line source with a current 1 A is positioned at the coordinate (10 *mm*, 0).

In fact, controlling radiation pattern is also realized in the aforementioned cavity structure. From Fig. 2(b), we can observe that dipole mode could be excited inside the cavity and radiate to free space when the working frequency is at the resonant frequency of dipole mode, which is about 12.167 GHz. While for other higher order modes, if the required resonant frequencies could be achieved, similar radiation effects could also be observed. For example, when the working frequency is about 16.33 GHz, it is corresponding to the resonance of cavity mode with *m* = 2. The dominant mode inside the cavity is quadrupole mode, hence the radiated EM wave spreads to four directions (see Fig. 4a). When we set the working frequency as about 20.29 GHz, the resonance of cavity mode with *m* = 3 occurs. The hexapole mode as a dominant mode is excited inside the cavity, as a result the radiated EM wave propagates to six directions, as shown in Fig. 4(b). Analogically, we can imagine that by choosing suitable working frequency, the resonance of any cavity mode could happen in principle, leading to a peculiar EM wave radiation pattern with numbers of outgoing direction determined by the angular momentum *m*.

### Achieving isotropic or directive radiation from a line source

**Figure 5:**
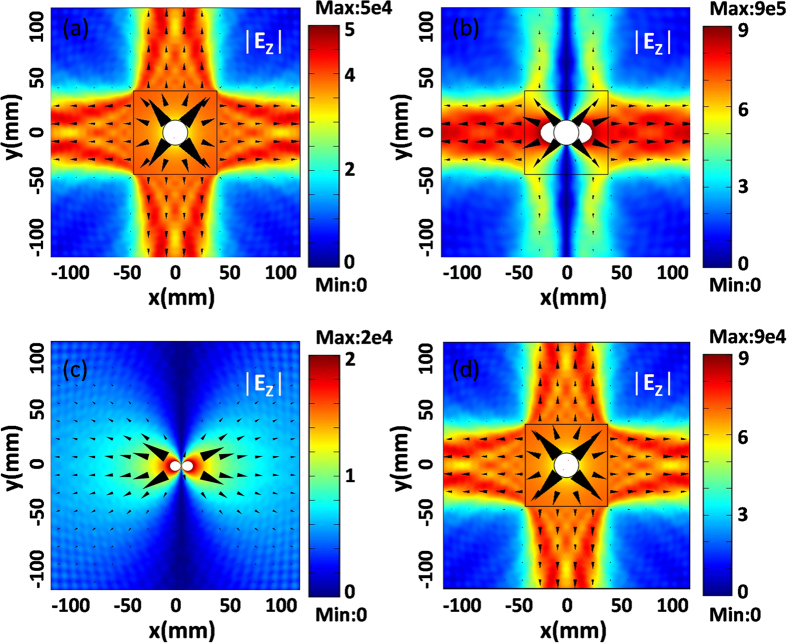
Simulated electric field distribution patterns for different types of sources. **(a)** The excited monopole mode by a line source inside the cavity. **(b)** The excited dipole mode by a line source inside the cavity. **(c)** A dipole source is placed in air. **(d)** The excited monopole mode by a dipole source inside the cavity. In plots a, b, d, the core regions are air with radii *R*_1_ = 15 *mm*. The squares with side length 100 *mm* represent the outer boundary of MNZ regions with parameters *ε*_2_ = 1 and *μ*_2_ = 0.001. A line source with a current 1 A is placed at the position (10 *mm*, 0) for case (**a**) and (**b**), while for case (**c**) and (**d**), the electric dipole source consists of two line currents of −1 A and +1 A and the line sources are placed at the positions (5 *mm*, 0) and (10 *mm*, 0) respectively. The white region means that the field values are beyond the scope of the color bar.

When a line source is placed in ZIMs, the radiation direction could be tailored by the external geometric shape of ZIMs^17^,^18^,^19^. For example, when a line source is placed inside ZIMs whose external shape is a square, it will generate four outgoing beams with radiation directions vertical to the four sides of the ZIM region. To verify such an interesting property from the proposed cavity structure, Fig. 5(a) shows the case in which the external circular shape of the ZIM shell in Fig. 1(b) is changed into a square one. In the simulations, the side length of the square is set as 100 *mm*, and the radius of the inner core region is still 15 *mm*. When the working frequency is about 17.63 GHz (almost corresponding to the resonant frequency of monopole mode in Supplementary Fig. S3), the resonance of monopole mode will occur inside the cavity as shown in Fig. 5(a). From the simulated electric field distribution pattern, we can find that the source generates four beams propagating into free space, almost vertical to the four sides of the ZIM region. It is because the monopole mode is excited inside the cavity to produce isotropic radiation and such radiation is tailored by the external square shape to bring about the four propagating beams. The energy flows marked by black arrows verify such directionality of radiation as well. When the working frequency is about 12.173 GHZ (a little shift from the resonant frequency of dipole mode in Fig. 2b), we observe that the dipole mode is excited inside the cavity (the dipole mode is not obvious, as the color bar is too small) and generates directive radiation as shown in Fig. 5(b), where most EM energy tends to *x* and –*x* directions and the energy is much weaker in other directions. Such a characteristic can be viewed again from radiated energy flows marked by the black arrows. Given that, though the external shape of ZIMs is changed, which will shift slightly the resonant frequencies of cavity modes, but the intrinsic cavity modes are intact. In other words, the intrinsic radiation, induced by the resonances of cavity modes, is independent of external geometric shapes.

### Obtaining isotropic radiation from a dipole source

As we know, an electric dipole source in air could produce directional radiation, as shown in Fig. 5(c). However, by placing the electric dipole source in the cavity structure, it could still generate isotropic radiation if the working frequency is at the resonant frequency of monopole mode. To prove this assumption, we set the frequency of dipole source as 17.63 GHz, which is the resonant frequency of monopole mode in Fig. 5(a). In fact, the monopole mode takes place in the cavity (please see Fig. 5d), but it is not clear because the field is beyond the color bar. However, from the electric field distribution pattern in Fig. 5(d), an isotropic radiation is indeed achieved by the electric dipole source. As a consequence, the radiation patterns, in fact, are independent of the types of sources (e.g., line current source or electric dipole source), and they only are determined by the excited cavity modes. In comparison to the field amplitude in Fig. 5(a) where only one line source is placed, the field amplitude in Fig. 5(d) is enhanced about twice. That is because we generate the electric dipole source with two line sources with opposite phases in Fig. 5(c,d). Hence, the cavity structure can also be used to realize power combination^32^,^33^. All in all, the proposed cavity structure exhibits much advantage on controlling EM radiation of sources, as compared with previous works^33^,^34^,^35^,^36^,^37^,^38^ in which different devices are based on special sources.

## Discussion

In this work, we demonstrate that when dipole mode is excited in a cylindrical cavity structure covered with MNZ, inhomogeneous field will happen in ZIMs. In fact, if the cavity modes except monopole modes are excited, they always induce inhomogeneous field in ZIMs even if the value of near zero parameter is very tiny (*i.e.,* 10^−7^≤*μ*_2_≤10^−3^). In addition, such cavity structure can be utilized to control the EM radiation of sources in versatile ways. We find that to enhance or to suppress EM radiation, it depends on the resonances of cavity modes. The peculiar radiation patterns are determined by the angular momentum number. In particular, the resonance of monopole mode leads to isotropic radiation, while the resonance of dipole mode gives rise to directive radiation. What’s more, such EM radiation induced by cavity modes could be tailored further by the external geometric shape of ZIMs and is independent of the types of sources. On the basis of these features, our study provides a new strategy to manipulate radiation of sources. Though we assume dispersionless MNZ metamaterials in this work, for a narrow band of MNZ, by filling required materials inside the core region, similar results could still be achieved (please see Supplementary Note 4).

## Additional Information

**How to cite this article**: Fu, Y. *et al.* Inhomogeneous field in cavities of zero index metamaterials. *Sci. Rep.* **5**, 11217; doi: 10.1038/srep11217 (2015).

## Supplementary Material

Supplementary Information
