# Corrigendum: Inhomogeneous field in cavities of zero index metamaterials

**DOI:** 10.1038/srep14021

**Published:** 2015-09-21

**Authors:** Yangyang Fu, Yadong Xu, Huanyang Chen

Scientific Reports 5: Article number: 1121710.1038/srep11217; published online: 06162015; updated: 09212015

The original version of this Article contained a typographical error in the spelling of the author Huanyang Chen, which was incorrectly given as Huangyang Chen. This has now been corrected in both the PDF and HTML versions of the Article.

