# Supplementary material for: Inhomogeneous field in cavities of zero index metamaterials
Source: Sci Rep. 2015 Jun 16;5:11217. doi: 10.1038/srep11217 (PMC4468819; doi:10.1038/srep11217)
Supplement: Supplementary Information [file srep11217-s1.pdf]

Supplementary Information for:

Inhomogeneous field in cavities of zero index metamaterials

Yangyang Fu, Yadong Xu & Huangyang Chen

College of Physics, Optoelectronics and Energy & Collaborative Innovation Center of Suzhou Nano Science and Technology, Soochow University,

No.1 Shizi Street, Suzhou 215006, China.

ydxu@suda.edu.cn or *chy@suda.edu.cn*

Supplementary Figures

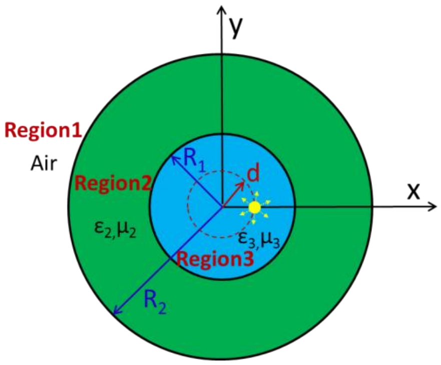

Supplementary Figure S1. A schematic plot of 2D cylindrical cavity structure in free space. Region 2 represents the shell of MNZ metamaterials, with its inner and outer radii R_1 and R_2 respectively. Inside the shell, it is the filled medium. An electric line source is positioned at a coordinate of (d, 0), as shown by the yellow point.

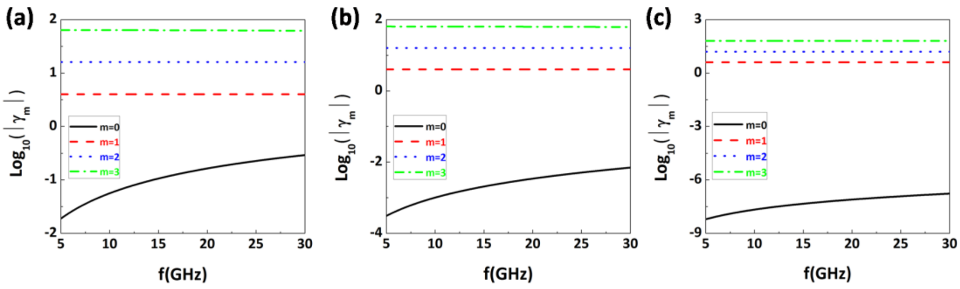

Supplementary Figure S2. The analytical result for the ratio γ_m **. (a), (b)** and **(c)**are the corresponding results for the ratio γ_m for the cases of $\mu_2 = 10^{-3}, 10^{-5}, 10^{-10}$ respectively. In plots, the black solid, red dashed, bule dot and green dash dot are the cases of the order m=0,1,2,3 for γ_m respectively.In all calculations, the related parameters are set as follows: R_1 = 15mm, R_2 = 30mm, d =10mm and $\varepsilon_2 = 1$. The core medium is air.

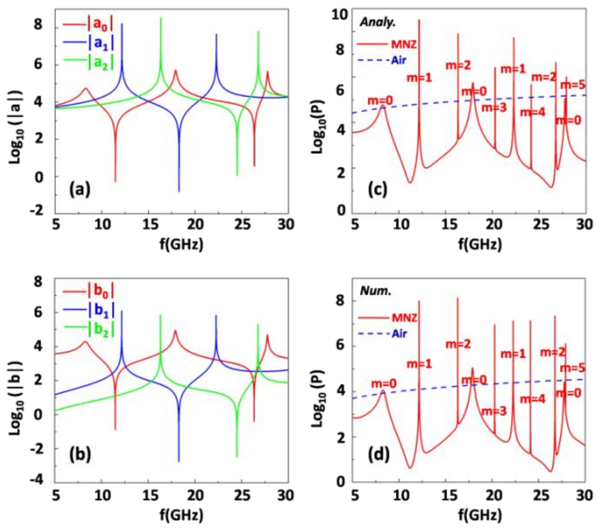

Supplementary Figure S3. The cofficients of excited modes and the power flowof radiated EM wave. (a) Analytical cofficients a_m for m = 0, 1 and 2. **(b)**Analytical cofficients b_m for m = 0, 1 and 2.**(c)**Analytical results of the power flow in free space. **(d)**Numerically calculated results of the power flow in free space. For (c) and (d), the red solid curve is the calculated power flow for the case of region 2 made of MNZ, while the blue dashed curve is the calculated power flow for the case of free space. The line source is with a current $I_S = 1$A. All the results are in a logarithmic scale. In all calculations, the related parameters are as follows: R_1 = 15*mm*, R_2 = 30*mm*, *d* =10*mm*, $\mu_2 = 10^{-3}$ and $\varepsilon_2 = 1$. The core medium is air.

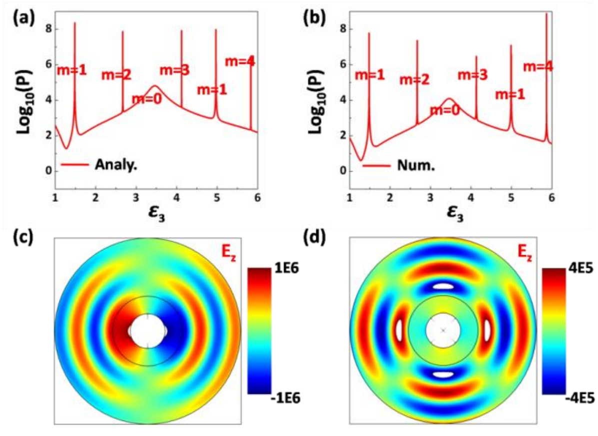

Supplementary Figure S4. Cavity modes of MNZ metamaterials by changing the dielectric constant of the core medium at 10GHz. **(a)**Analytical results of the power flow in free space. **(b)**Numerically calculated results of the power flow in free space.**(c)**The real part of electric field for dipole mode resonance.**(d)** The real part of electric field for quadrupole mode resonance. For case (c) and (d), the corresponding permittivity of the filled media in the core region are about $\varepsilon_3 = 1.48, \varepsilon_3 = 2.67$ respectively. In all calculations, the related parameters are as follows: R_1 = 15*mm*, R_2 = 30*mm*, *d* =10*mm*, $\mu_2 = 10^{-3}$, $\mu_3 = 1$ and $\varepsilon_2 = 1$.

Supplementary Notes

Supplementary Note 1

Theoretical analysis

The two-dimensional (2D) cylindrical cavity structure is placed in free space, as shown schematically in Supplementary Fig.S1. The shell with MNZ metamaterials is uniform in the *z* direction, with its inner and outer radii R_1 and R_2 respectively. Suppose that the effective permittivity and permeability of MNZ are ε_2 and μ_2 respectively. For the core region, the parameters of filled medium are denoted as ε_3 and μ_3. An infinite electric line source is located at a position of (*d*, 0), as indicated by the yellow point in Supplementary Fig.S1. Under 2D cylindrical coordinate system denoted by r, θ and *z*, only three components E_z, H_r and H_θ are involved, and the EM wave in each region is governed by Helmholtz equation:

$$\frac{\partial}{\partial r}\left(r\frac{\partial E_z}{\partial r}\right)+\frac{1}{r}\frac{\partial^2 E_z}{\partial \theta^2}+\frac{\omega^2}{c^2}\varepsilon_N\mu_N rE_z=0\,, \tag{S1}$$

where ω is the angular frequency, and *c* is velocity of light in vacuum, and *N* = 1, 2 and 3, labeling three different regions. By analyzing equation (1), we can obtain the general solution E_z in each region as follows:

in region 1,

$$E_z^{(1)}=\sum_{m=-\infty}^{+\infty} b_m H_{\mathrm{m}}(k_0 r)e^{im\theta},\ r\geq R_2\,, \tag{S2}$$

and in region 2,

$$E_z^{(2)}=\sum_{m=-\infty}^{\infty}\left[c_m H_{\mathrm{m}}(k_2 r)+d_m J_m(k_2 r)\right]e^{im\theta},\ R_1\leq r<R_2\,, \tag{S3}$$

where $J_m(x)$ and $H_m(x)$ are the *m-th* order of Bessel function and Hankel function of the first kind respectively, and $k_0=\omega/c$ is the wave vector in vacuum or air, and $k_2=k_0\sqrt{\varepsilon_2\mu_2}$ is the wave vector in MNZ of region 2, and b_m, c_m and d_m are the unknown coefficients to be determined. For simplicity, the time variation $e^{-i\omega t}$ is omitted throughout this work.

For region 3, the EM wave should be the superposition of all the cavity modes and the source radiation. Generally, the field distribution produced by an infinite line source is an angular-independent uniform cylindrical wave which is described by the zero-*th* order Hankel function of first kind, *i.e.*, $E_z^p=\alpha H_0(k_3\rho)$, where $\alpha=-\frac{1}{4}\omega\mu_0 I_S$ is a coefficient related to the source itself, and $k_3=k_0\sqrt{\varepsilon_3\mu_3}$ is the wave vector in region 3 and ρ is the radial coordinate under another cylindrical coordinate whose origin is centered at the source. In order to analyze conveniently, it is necessary to expand such afield distribution under the coordinate with its origin at the center of region 3. By applying the translational addition theorem, we have,

$$E_z^p=\alpha H_0(k_3\rho)=\begin{cases}\displaystyle\sum_{m=-\infty}^{\infty}\alpha J_m(k_3 d)e^{-im\phi}H_m(k_3 r)e^{im\theta},\ r\geq d\\ \displaystyle\sum_{m=-\infty}^{\infty}\alpha H_m(k_3 d)e^{-im\phi}J_m(k_3 r)e^{im\theta},\ r<d\end{cases}, \tag{S4}$$

where $\cos\phi=\vec{d}\cdot\hat{x}/d$ is the cross angle between translation vector $\vec{d}$ and *x*-axis. For a source at

coordinate (d, 0), it has $\phi = 0$. Thereby, the total field distribution in region 3 is expressed as:

$$E_z^{(3)} = \sum_{m=-\infty}^{\infty} \left[p_m H_m(k_3 r) + a_m J_m(k_3 r) \right] e^{im\theta},\ d \le r \le R_1, \tag{S5}$$

where $p_m = \alpha J_m(k_3 d)$ is a constant, which is dependent on the source and its position, a_m is the unknown coefficient, determining the amplitude of excited cavity modes inside region 3. It is noted that in all regions, the distributions of corresponding magnetic fields could be obtained from $\vec{H} = \nabla \times \vec{E} / (i\omega\mu_0\mu_N)$.

Before inspecting the radiation of the cavity, let us discuss two situations about the position of the source inside region 3. (A) The source is located at the center of the cavity, which means that $d = 0$. For this case, from the expressed field distribution of equation (S5), we can see that the coefficient of the source is $p_m = 0$ for any order m excepting the zero order, stemming from Bessel functions $J_0(0) = 1$ and $J_{m\neq 0}(0) = 0$. Under such circumstance, there is only a_0 left for the coefficients a_m, while other a_m ($m \neq 0$) are null because the source does not radiate energy of higher order modes. It indicates that only monopole mode could be excited inside the cavity, resulting in only omni-directional or isotropic radiation in free space. (B) The source is deviating from the center, that is $d \neq 0$. In this case, the coefficient p_m becomes meaningful for any order m. Thereby, all cavity modes in region 3 could be excited in principle, which makes the physics inside the cavity more interesting. In the following, we will focus on the latter case. To obtain all unknown coefficients a_m, b_m, c_m and d_m, we apply the continuous boundary conditions of tangential fields, *i.e.* E_z and H_θ, at interfaces $r = R_2$ and $r = R_1$. After some mathematical derivations, we could obtain,

$$a_m = -\frac{gH'_m(k_3R_1) - A_m H_m(k_3R_1)}{gJ'_m(k_3R_1) - A_m J_m(k_3R_1)} p_m, \tag{S6a}$$

$$b_m = \frac{p_m H_m(k_3R_1) + a_m J_m(k_3R_1)}{B_m H_m(k_0R_2)}, \tag{S6b}$$

$$c_m = D_m b_m, \tag{S6c}$$

$$d_m = C_m c_m, \tag{S6d}$$

with $A_m = \dfrac{H'_m(k_2R_1) + C_m J'_m(k_2R_1)}{H_m(k_2R_1) + C_m J_m(k_2R_1)}$, $B_m = \dfrac{H_m(k_2R_1) + C_m J_m(k_2R_1)}{H_m(k_2R_2) + C_m J_m(k_2R_2)}$,

$$C_m = -\frac{\eta_2 H_m(k_2R_2) H'_m(k_0R_2) - H_m(k_0R_2) H'_m(k_2R_2)}{\eta_2 J_m(k_2R_2) H'_m(k_0R_2) - H_m(k_0R_2) J'_m(k_2R_2)},$$

$$D_m = \frac{H_m(k_0R_2) J'_m(k_2R_2) - \eta_2 J_m(k_2R_2) H'_m(k_0R_2)}{H_m(k_2R_2) J'_m(k_2R_2) - J_m(k_2R_2) H'_m(k_2R_2)}$$

$g = \eta_2 / \eta_3$ and $\eta_{2(3)} = \sqrt{\mu_{2(3)} / \varepsilon_{2(3)}}$.

where the prime in functions $H'_m(x)$ and $J'_m(x)$ represents their derivations.

Supplementary Note 2

For the ratio γ_m

As we have obtained all the unknown coefficients, the detailed field distributions in all the regions should also be obtained. For the field in region 2, we can optimize it (that is, the Eq.(S3)) by defining the ratio $\gamma_m = c_m H_\mathrm{m}(k_2 r) / d_m J_m(k_2 r)$ where we select $r = R_1$ for γ_m. We find that, for m=0, $\gamma_0 \ll 1$, while for $m \neq 0$, $\gamma_m \gg 1$, regardless of the value of near zero permeability μ_2 (see Supplementary Fig.S2). Hence, the electric field in region 2 could be written as,

$$E_z^{(2)} = d_0 J_0(k_2 r) + \sum_{m=-\infty, m\neq 0}^{\infty} c_m H_\mathrm{m}(k_2 r) e^{im\theta}, \ R_1 \leq r < R_2 . \tag{S7}$$

When one of the cavity modes is at resonance, such mode is the most dominative one. Then Eq.(S7) could be approximately expressed as,

$$E_z^{(2)} = d_0 J_0(k_2 r) + c_m H_\mathrm{m}(k_2 r) e^{im\theta}, \ R_1 \leq r < R_2 , \tag{S8}$$

where $m \neq 0$.

Supplementary Note 3

Radiation from the cavity

In fact, the two coefficients a_m and b_m, related to the excited cavity modes inside region 3 and the radiated EM wave in free space respectively, are enough for us to fully uncover the radiation issue. Based on the above analytical formulas, Supplementary Fig.S3(a) shows the relationship between coefficients a_m *vs* frequencies, while Supplementary Fig.S3(b) reveals the coefficients b_m *vs* frequencies. In this analysis, the permeability and permittivity of MNZ metamaterials are set as $\mu_2 = 10^{-3}$ and $\varepsilon_2 = 1$ respectively. The geometric sizes of the cavity are set as R_1 = 15*mm* and R_2 = 30*mm*, and the core medium is air. For the source, we set $I_S = 1A$ and d = 10*mm*. For simplicity, we just display three orders with m = 0, 1 and 2 in plots, which are corresponding to monopole (in red), dipole (in blue) and quadrupole modes (in green) respectively. The three different coefficients a_m is clearly observed in Supplementary Fig.S3(a), *e.g.* for dipole mode shown by the blue curve, there are several peaks attributed to the resonances of the related cavity modes. Compared to monopole modes, the resonances of higher order modes are much sharper. The main reason is the intrinsic properties of ZIMs. The MNZ shell is transparent for monopole modes inside the cavity, while it is opaque for all the others. Besides these peaks, we can also observe some resonant dips, and their resonant frequencies satisfy such a condition: $p_m = \alpha J_m(k_3 d) = 0$. It means that at such frequencies, the excitations of corresponding modes are greatly suppressed. Moving to the radiated coefficients b_m, there are similar resonant peaks and dips as shown in Supplementary Fig.S3(b). For the resonant peaks of b_m, they are resulted from the resonances of cavity modes, hence both resonances for a_m and b_m share the same resonant frequencies. While for the resonant dips of b_m, they are due to the suppression of the cavity modes. As a result, a_m and b_m have an identical resonant dip condition given by $p_m = \alpha J_m(k_3 d) = 0$. Furthermore, by comparing coefficients a_m and b_m in Supplementary Fig.S3(a) and Fig.S3(b) respectively, we can find the amplitudes of b_m are less than these of a_m ,which is caused by impendence mismatching between the MNZ shell and air.

Enhancing or suppressing radiation

In order to exhibit intuitively the total EM energy radiating from the cavity structure, we need to compute analytically the summation of the power flow of all modes from $m=-\infty$ to ∞, by using $P=\frac{1}{2\eta_0}\left|E_z^{(1)}\right|^2=\frac{1}{2\eta_0}\sum_m\left|b_m H_m(k_0R_2)\right|^2$, where η_0 is impedance of air. However, in a selected frequency range (*e.g.*, 5 GHz to 30 GHz), the number of possible resonant cavity modes are finite. Therefore, we choose the required order from *m*=-5 to 5 to calculate the power flow in such a frequency range, which is also enough to make the result convergent to an exact value. Supplementary Fig.S3(c) shows such an analytical result of the power flow, where there are sets of resonant peaks. Compared to the positions of such peaks with those in Supplementary Fig.S3(b), obviously, all radiation resonances are resulted from the resonances of each cavity modes which is clarified by the order *m* one by one in plot. By observing all peaks in Supplementary Fig.S3(c), we can find that the bandwidths of monopole radiation are much broader, while the resonant peaks of higher order modes ($m \neq 0$) are quite sharp. Such sharp resonances have great applications among optical sensors or detecting systems. Moreover, with COMSOL Multiphysics, Supplementary Fig.S3(d) presents the numerically calculated power flow radiating from the cylindrical cavity. In simulations, we choose the same cavity structure and material parameters as described previously. The numerical results match the analytical results quite well. In particular, all resonant positions in both results are almost coincident. By observing the results of Supplementary Fig.S3(c) and (d), we can find that when the resonance of cavity modes are obtained, *i.e.*, at the resonant peaks, the enhancing EM radiation could be achieved, otherwise the EM radiation will be suppressed.

Supplementary Note 4

Cavity modes in a narrow band of MNZ

In the main text, we assume a dispersionless MNZ metamaterials in order to conveniently and clearly explain the potential physics of inhomogeneous field in the cavity structure. However, by changing the filled media in region 3, similar results could be obtained. For example, we assume that the working frequency of MNZ is at 10 GHz, and by changing filled media in the core region, we can still observe the corresponding resonances of cavity modes, as shown in Supplementary Fig.S4. Supplementary Fig.S4(a) shows the analytical results of power flow radiating from the cavity and the enhancing EM energy could be obtained when they are at resonance of cavity modes, which are marked by the order *m* one by one in plot. The corresponding numerical result is displayed in Supplementary Fig.S4(b), which matches well with the analytical one. In addition, when the required media are filled in core region 3, the resonance of cavity modes will occur. Especially, when the higher order modes are at resonance, the inhomogeneous field will happen in ZIMs. Supplementary Fig.S4(c) shows the real part of the electric field for the case of dipole mode resonance, and we can read the information of inhomogeneous field in ZIMs. Supplementary Fig.S4(d) shows the real part of the electric field , where the quadrupole mode is at resonance. The inhomogeneous field in ZIMs could still be observed, and the radiation pattern with numbers of outgoing directions determined by the angular momentum *m*=2. Therefore, although MNZ metamaterials usually work for a narrow band of frequencies, if the required media are filled in the core region, all similar results could be achieved.
